# Control of Ca^2+^ Influx and Calmodulin Activation by SK-Channels in Dendritic Spines

**DOI:** 10.1371/journal.pcbi.1004949

**Published:** 2016-05-27

**Authors:** Thom Griffith, Krasimira Tsaneva-Atanasova, Jack R. Mellor

**Affiliations:** 1 Department of Engineering Mathematics, University of Bristol, Bristol, United Kingdom; 2 Bristol Centre for Complexity Sciences, University of Bristol, Bristol, United Kingdom; 3 Department of Mathematics, College of Engineering, Mathematics and Physical Sciences, University of Exeter, Exeter, United Kingdom; 4 EPSRC Centre for Predictive Modelling in Healthcare, University of Exeter, Exeter, United Kingdom; 5 Centre for Synaptic Plasticity, School of Physiology, Pharmacology and Neuroscience, University of Bristol, Bristol, United Kingdom; The Krasnow Institute for Advanced Studies, UNITED STATES

## Abstract

The key trigger for Hebbian synaptic plasticity is influx of Ca^2+^ into postsynaptic dendritic spines. The magnitude of [Ca^2+^] increase caused by NMDA-receptor (NMDAR) and voltage-gated Ca^2+^ -channel (VGCC) activation is thought to determine both the amplitude and direction of synaptic plasticity by differential activation of Ca^2+^ -sensitive enzymes such as calmodulin. Ca^2+^ influx is negatively regulated by Ca^2+^ -activated K^+^ channels (SK-channels) which are in turn inhibited by neuromodulators such as acetylcholine. However, the precise mechanisms by which SK-channels control the induction of synaptic plasticity remain unclear. Using a 3-dimensional model of Ca^2+^ and calmodulin dynamics within an idealised, but biophysically-plausible, dendritic spine, we show that SK-channels regulate calmodulin activation specifically during neuron-firing patterns associated with induction of spike timing-dependent plasticity. SK-channel activation and the subsequent reduction in Ca^2+^ influx through NMDARs and L-type VGCCs results in an order of magnitude decrease in calmodulin (CaM) activation, providing a mechanism for the effective gating of synaptic plasticity induction. This provides a common mechanism for the regulation of synaptic plasticity by neuromodulators.

## Author Summary

Hebbian or associative plasticity is triggered by postsynaptic Ca^2+^ influx which activates calmodulin and CaMKII. The influx of Ca^2+^ through voltage-dependent NMDA receptors and Ca^2+^ channels is regulated by Ca^2+^ -activated K^+^ channels (SK-channels) providing negative feedback regulation of postsynaptic [Ca^2+^]. Using 3-dimensional modeling of Ca^2+^ and calmodulin dynamics within dendritic spines we show that the non-linear relationship between Ca^2+^ influx and calmodulin activation endows SK-channels with the ability to “gate” calmodulin activation and therefore the induction of Hebbian synaptic plasticity. Since SK-channels are inhibited by several neuromodulator receptors including acetylcholine and noradrenaline, the gating of synaptic plasticity by SK-channels could represent a common mechanism by which neuromodulators control the induction of synaptic plasticity.

## Introduction

Associative learning is underpinned by Hebbian synaptic plasticity at glutamatergic synapses. Spike timing-dependent plasticity (STDP) is the classical manifestation of Hebbian plasticity where temporally correlated pre- and post-synaptic activity induces NMDA receptor (NMDAR)- and Ca^2+^ -dependent changes in synaptic strength [1–3]. The calcium control hypothesis states that the direction of synaptic plasticity is determined by the amplitude of postsynaptic [Ca^2+^] transients; moderate elevations of [Ca^2+^] result in long-term depression (LTD), whereas higher levels of [Ca^2+^] lead to long-term potentiation (LTP) [4–6]. However, due to high spine-neck electrical resistance [7, 8], large amplitude [Ca^2+^] transients can be elicited by presynaptic stimulation in the absence of postsynaptic spiking without inducing synaptic plasticity [7, 9] raising a question mark over the validity of the calcium hypothesis. Furthermore, the requirement for Ca^2+^ influx through voltage-gated Ca^2+^ channels (VGCCs) and temporally precise postsynaptic spiking [9–11] highlights the importance of spatiotemporal patterning of [Ca^2+^] within the postsynaptic spine for induction of STDP, although the mechanisms for this remain obscure.

The expression of Hebbian synaptic-plasticity is dependent on activation of Ca^2+^ /calmodulin-activated kinase II (CaMKII) and therefore on the transduction of Ca^2+^ signals by calmodulin (CaM) [12, 13]. Interestingly, activation of CaM by Ca^2+^ does not follow a linear relationship because CaM activation requires Ca^2+^ binding to sites at both the C- and N-terminals of CaM, which have different affinities and kinetics for Ca^2+^ binding. The C-terminal lobe is high affinity with slow binding kinetics whilst the N-terminal lobe is low affinity with fast binding kinetics [14]. This ensures that CaM activation by Ca^2+^ within spines will depend on the spatiotemporal pattern of [Ca^2+^] transients. In turn, spatiotemporal [Ca^2+^] distributions are determined by endogenous Ca^2+^ buffering within spines which restrict Ca^2+^ to localized micro- or nano-domains [15]. Further control of spine Ca^2+^ dynamics is provided by small-conductance Ca^2+^ -activated K^+^ channels (SK-channels) located in the spine membrane [16, 17]. SK-channels are activated by Ca^2+^ influx through NMDARs and VGCCs. The resulting hyperpolarization and inhibition of these voltage-dependent channels constitutes a negative regulatory feedback mechanism on Ca^2+^ influx. Conversely, inhibition of SK-channels relieves the negative regulation resulting in greater spine depolarization and Ca^2+^ influx which facilitates the induction of LTP [16, 18] and enhances performance in spatial memory tasks [19]. It is therefore predicted that SK-channels regulate spine Ca^2+^ dynamics which at the nanodomain-level control CaM activity and the induction of synaptic plasticity.

These signaling nanodomains are beyond the resolution of currently available microscopy techniques therefore to test the predicted nonlinear-feedback effects of SK-channels on spine Ca^2+^ and CaM we developed a 3-dimensional, deterministic reaction-diffusion model within a biophysically plausible dendritic spine calibrated to experimentally recorded global spine-Ca^2+^ transients from CA1 pyramidal neurons in the hippocampus. Using this model we qualitatively addressed the effect of the SK-channel interactions with spine Ca^2+^ and CaM, and considered the implications for synaptic plasticity induction. We find that during STDP, SK-channels are activated in response to a priming Ca^2+^ stimulus from either NMDARs or VGCCs. The subsequent reduction in Ca^2+^ influx through NMDARs and L-type VGCCs results in a order of magnitude decrease in CaM activation providing a mechanism for the effective gating of synaptic plasticity induction.

## Methods

Solutions to the spine Ca^2+^ diffusion model were obtained using finite-element methods implemented in COMSOL Multiphysics 4.2a using LiveLink for MATLAB [20, 21]. Full model equations are given in the Supporting Information. Model parameter-values were constrained using experimental data from electrophysiological and Ca^2+^ imaging studies, performed on hippocampal CA1 dendritic spines where possible (S1 Table). For spine morphology, we used a solution domain represented by the union of a sphere for the head, and a cylinder for the spine neck. Volume boundaries outside of channel cluster regions had zero-flux condition. Channel cluster boundary regions had time-varying Ca^2+^ flux conditions determined by a Hodgkin-Huxley-type model formulation. Spine head and neck dimensions were matched to the median of reported CA1 spine size-distributions [8]. The boundary of the spine was discretized into triangular elements and the volume within the boundary was discretized into tetrahedral elements. Predefined mesh parameter values were set to Normal (maximum and minimum element size 0.1*μ*m and 0.02*μ*m, respectively) in the interior of the spine, and Extremely Fine (maximum and minimum element size 0.02*μ*m and 0.0002 *μ*m, respectively) within 50nm of Ca^2+^ sources. The time-dependent solver used implicit time-stepping backward differentiation formula (BDF) scheme (maximum BDF order 2) with adaptive time-step (maximum allowable step, 10*μ*s). Scaled absolute tolerance for solution variables was 0.001. Each ion-channel cluster was represented by a circular region (diameter 1nm) on the boundary. Ca^2+^ flux boundary conditions for NMDAR and VGCC clusters varied in time according to the current densities calculated by the model. Ion channels included were; AMPARs (total maximal conductance, *g*_A_ = 60 pS), NMDARs (*g*_N_ = 160 pS), T-type (*g*_Ca_T__ = 0.23 pS) and L-type VGCCs (*g*_Ca_L__ = 0.9 pS), and SK-channels (*g*_SK_ = 25 pS). AMPAR and NMDAR open probabilities were modeled using a glutamate binding-scheme [22]. The NMDAR open probability also incorporated Mg^2+^-unblock voltage-dependence. Activation and inactivation gating-variable steady-states for T-type and L-type VGCCs were modeled using equation form described in [23]. The SK-current was governed by a Hill function which was dependent on local [Ca^2+^] to the SK-channel cluster. EPSPs were simulated by representing synaptic-cleft glutamate concentration immediately after presynaptic stimulus as a step-pulse of 1mM amplitude with duration 1ms. bAPs were simulated by instantaneous rise of membrane potential to maximal depolarization (67mV), followed by double exponential-decay to resting potential. Diffusing Ca^2+^ was buffered by a low binding-capacity endogenous fixed-buffer (EFB) (binding ratio of 20 [24]) and a mobile buffer (unless otherwise stated, 100*μ*M calbindin, binding ratio ≈ 250, *D*_*B*_ = 20 *μ*m^2^s^−1^). In simulations involving calmodulin, the simple mobile-buffer model (Eq. 16) was replaced with a cooperative binding model of Ca^2+^ to the two calmodulin lobes [25] (S1 Fig). Unless otherwise stated, initial calmodulin concentration was set to 100*μ*M (effective binding ratio ≈ 35) and diffusivity to 20 *μ*m^2^s^−1^. Spine Ca^2+^ -extrusion mechanisms, including membrane pumps, exchangers and uptake into intracellular Ca^2+^-stores, were uniformly modeled throughout the spine volume by a single term, linearly-dependent on local [Ca^2+^]. Parameter values, constrained by Ca^2+^ -imaging experiments, were tuned (in simulations using mobile-buffer binding kinetics for the relevant Ca^2+^ -indicator dye) by matching changes in model-predicted levels of Ca^2+^ -bound mobile buffer to changes in measured Ca^2+^ dye fluorescence.

## Results

### 3-dimensional modeling of [Ca^2+^] dynamics in a dendritic spine

**Fig 1:**
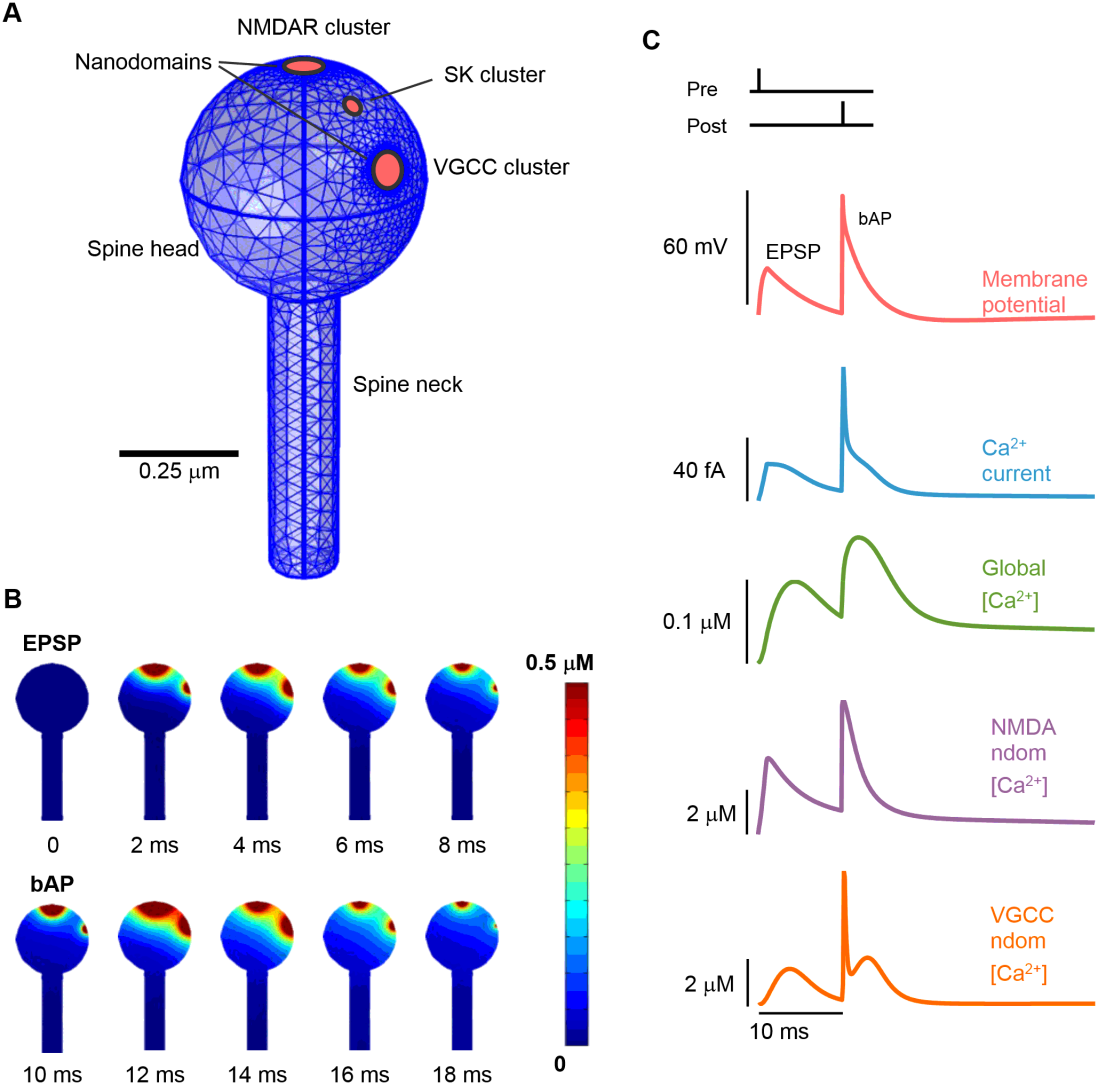
Modeling spine Ca^2+^ dynamics during STDP. *(A)* The spine is represented by the union of a sphere and a cylinder, with discrete NMDAR and VGCC channel cluster regions on the boundary. Ca^2+^ influx through these sources is regulated by local [Ca^2+^] at the SK-channel cluster. Channel nanodomains are indicated by red regions. *(B)* 2-dimensional cut-slice representation of [Ca^2+^] time-progression in the spine during an EPSP-bAP stimulation protocol. High [Ca^2+^] is confined to Ca^2+^ source vicinity. *(C)* Time courses of membrane potential and Ca^2+^ dynamics during the EPSP-bAP stimulation protocol. Ca^2+^ current (blue trace) is total Ca^2+^ current from all Ca^2+^ sources. Nanodomain [Ca^2+^] (purple and orange traces) is defined as the Ca^2+^ concentration at a radial distance of 20nm from the relevant Ca^2+^ source.

To test the hypothesis that spatiotemporal variation of [Ca^2+^] in postsynaptic dendritic spines is an important factor governing synaptic plasticity, we built a 3-dimensional, deterministic model of [Ca^2+^] dynamics within a single dendritic spine (Fig 1). Our approach is suited to Ca^2+^ simulation where Ca^2+^ influx at the boundary is dependent on multiple highly non-linear interactions evolving on multiple time scales and involving membrane potential and the [Ca^2+^] within spine nanodomains. Therefore stochastic effects were not included in the model. The biophysical properties of the modelled spine were estimated from experimental data and, where necessary, tuned by comparison to experimental spine Ca^2+^ responses to single bAPs or EPSPs (S1 Table).

The primary sources of Ca^2+^ influx in dendritic spines are NMDARs and VGCCs [26, 27]. NMDAR ion-channels tend to cluster at the post-synaptic density (PSD) and VGCCs are more evenly distributed in the spine membrane but are rarely found within 60nm of the PSD [28]. Due to this separation, and to facilitate our analysis of SK-channel coupling to these channels, we considered NMDARs and VGCCs as two distinct channel clusters in our model (Fig 1A). Within the VGCC cluster we included two subtypes of VGCC; low-voltage activated (LVA) T-type VGCCs and high-voltage activated (HVA) L-type VGCCs. SK-channels co-locate with both NMDARs and VGCCs [17, 29, 30] so whilst the locations of the NMDAR and VGCC channel clusters remained fixed for all simulations, we varied the position of the SK-channel cluster in some simulations in order to investigate the effects of SK-channel location relative to the main Ca^2+^ sources.

Another potential source of Ca^2+^ in dendritic spines is IP3-mediated or calcium-induced calcium release (CICR) from internal calcium stores. However, for CA1 spines, evidence suggests a limited role for calcium stores [31] (but see [32]), and so in line with similar modeling studies of spines at this synapse we have omitted calcium stores from our model [33, 34].

We characterized the model using the canonical STDP protocol for LTP—an EPSP followed by a bAP with 10ms delay (EPSP-bAP). Application of this protocol rapidly established a [Ca^2+^] gradient across the spine head with highest [Ca^2+^] near the Ca^2+^ channel clusters (Fig 1B). [Ca^2+^] did not equilibrate across the spine volume due to the rapid extrusion of Ca^2+^ (extrusion rate, *γ* = 5000s^−1^) (Fig 1B). The spatial extent of the [Ca^2+^] signal varied in accordance with the time course of the Ca^2+^ current (Fig 1B and 1C). EPSP magnitude was ∼25mV, in agreement with reported estimates for local spine-depolarizations [7, 35], and was large enough to generate Ca^2+^ currents through both NMDARs and LVA VGCCs [17], causing a transient increase in nanodomain [Ca^2+^] (Fig 1C). The bAP generated a distinct spiked-component of the VGCC-nanodomain [Ca^2+^] transient by activating the HVA VGCCs, and also caused a second peak in the NMDAR-nanodomain [Ca^2+^] transient by enhancing the NMDAR conductance via voltage-dependent Mg^2+^-unblock (Fig 1C). The global, or volume-averaged, spine [Ca^2+^] was an order of magnitude smaller in amplitude and temporally-smoothed in comparison to the nanodomain [Ca^2+^] time profiles. The global spine [Ca^2+^] peaked at around 0.15*μ*M, within the range of experimental estimates [24].

**Fig 2:**
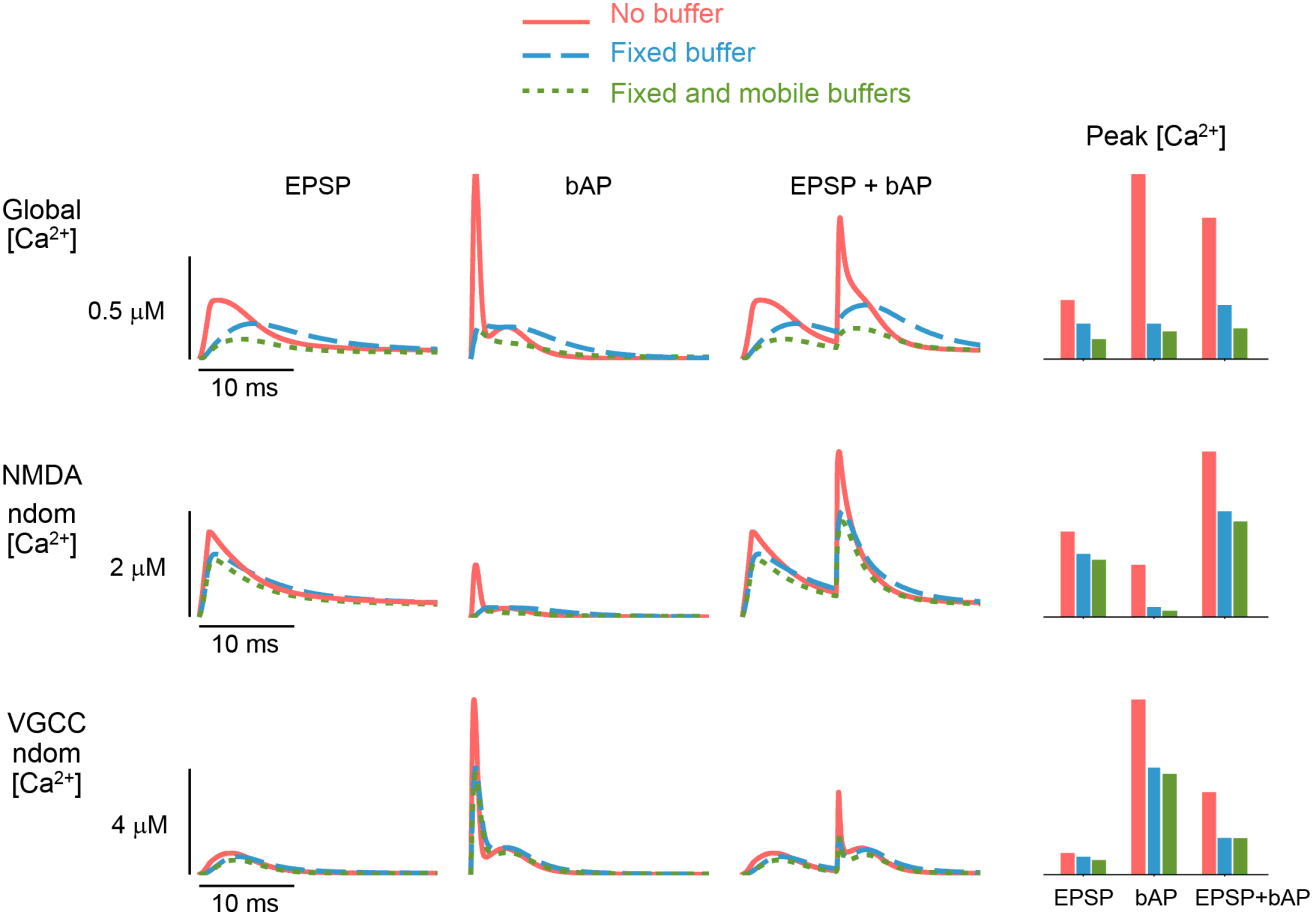
Low-pass filtering of global [Ca^2+^] signal by endogenous Ca^2+^ buffering. Global and nanodomain [Ca^2+^] transients for different Ca^2+^ buffering conditions during three different stimuli. Global [Ca^2+^] transients are temporally-smoothed by addition of Ca^2+^ buffers (top row). Smoothing effect on [Ca^2+^] transients is less pronounced in the channel nanodomains, defined here as [Ca^2+^] at 50nm radial distance from channel clusters (bottom two rows). Endogenous Ca^2+^ buffering prevents cross-talk between nanodomains, as demonstrated during a single bAP by the reduction of [Ca^2+^] signal in the NMDAR nanodomain (middle row, second panel). Bar plots provide a summary of [Ca^2+^] signal amplitudes across domains and buffer conditions (end column).

[Ca^2+^] -signal magnitude, rise and decay time, and spatial extent are perturbed by Ca^2+^ buffer changes [36]. When Ca^2+^ buffers with fast binding rates are present in high concentrations, large [Ca^2+^] signals are restricted to channel nanodomains [15]. Modeling studies examining steady-state solutions to the Ca^2+^ reaction-diffusion system have shown that at distances where the mean diffusion time for Ca^2+^ (from source) is less than the mean reaction time with Ca^2+^ buffers, endogenous buffers do not modify nanodomain [Ca^2+^] [37]. We investigated these effects, under transient boundary-flux conditions, by simulating three different stimuli across three Ca^2+^-buffering scenarios: 1) no buffer present, 2) endogenous fixed buffer (EFB) present, and, 3) both EFB and a mobile buffer (100*μ*M calbindin) present, the latter representing the most physiologically realistic scenario (Figs 2 and S2). Addition of endogenous buffers reduced the amplitude of the global [Ca^2+^] signal across all stimuli, but the relative changes in peak [Ca^2+^] were dependent on the stimulation type (Fig 2). [Ca^2+^] -transients with fast kinetics—such as the spiked bAP-elicited global [Ca^2+^] transient—were almost eliminated by temporal smoothing due to endogenous Ca^2+^ buffers. This filtering was much less effective in the NMDAR and VGCC nanodomains (Fig 2) due to the small time window for buffers to act on the diffusing Ca^2+^ in this domain [37, 38]. This was true for a range of endogenous Ca^2+^ buffers with physiologically plausible Ca^2+^ -binding kinetics and affinities (S3 Fig). From the perspective of the global domain, endogenous Ca^2+^ buffers act as a low-pass filter on [Ca^2+^] signals, whereas in channel nanodomains, [Ca^2+^] signals more faithfully track the time course of that channel-cluster’s Ca^2+^-current.

### SK-channel coupling to Ca^2+^ sources

**Fig 3:**
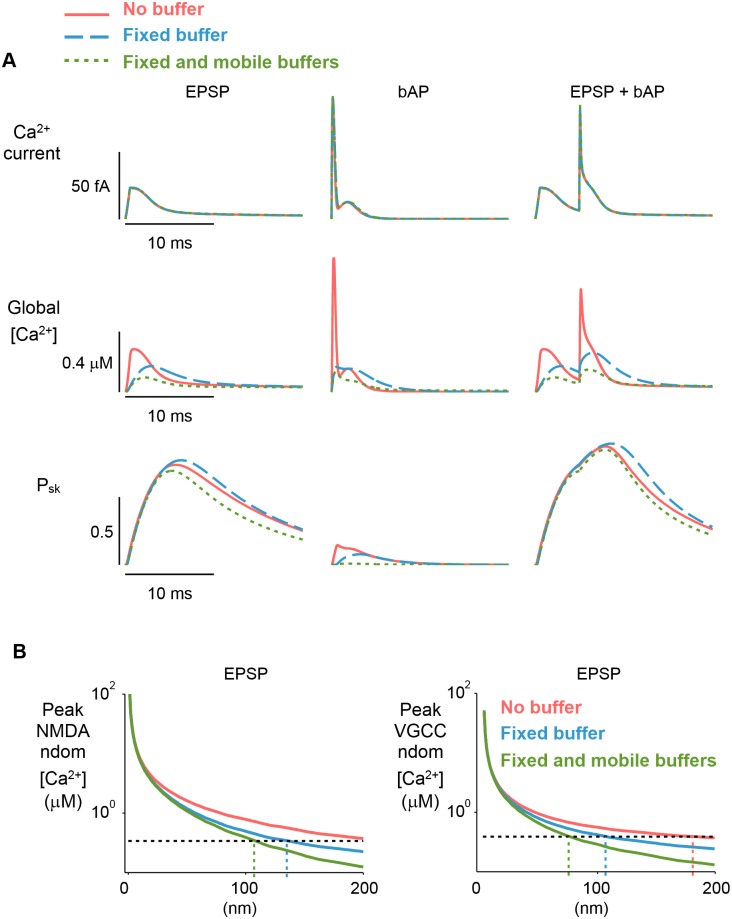
Tight-coupling of SK-channels to Ca^2+^ source ensures robust SK-channel activation. *(A)* With SK-channel location fixed at 50nm from NMDAR cluster, Ca^2+^ current is insensitive to buffer condition (top row). Global [Ca^2+^] transient amplitudes (middle row) are reduced by addition of endogenous buffers. Ca^2+^ current is unaffected by addition of endogenous buffers whereas SK-channel activation is weakly dependent on buffer condition but not such that it modulates the influx of Ca^2+^ (top and bottom rows). *(B)* Assuming SK-channel half-activation, *K*_s_ = 0.33*μ*M [40] (indicated by horizontal dashed line on plots), SK-channels with a 50nm coupling distance will be robustly activated in all three buffer conditions.

SK-channels are activated by Ca^2+^ influx through either NMDARs or VGCCs. The coupling strength of an SK-channel to a Ca^2+^ source in the spine, is determined by their distance and Ca^2+^ buffering [16, 39]. Therefore, we next tested the dependence of SK-channel activation (and the resulting regulation of Ca^2+^ influx) on SK-channel location and on Ca^2+^ buffering during three different stimuli. Initially, we fixed the SK-channel cluster location at a 50nm distance from the NMDAR cluster. For all stimuli, Ca^2+^ current was unaffected by changes to Ca^2+^ buffering, however, as shown previously (Fig 2), peak global [Ca^2+^] was reduced (Fig 3A). This confirmed that the sensitivity of the [Ca^2+^] transient to Ca^2+^ buffering condition was due to the direct action of buffers on Ca^2+^ rather than a more complex interaction involving buffers and [Ca^2+^] local to the SK-channel cluster.

SK-channel to Ca^2+^ source coupling distances of <∼100nm produced robust SK-channel activation during an EPSP regardless of buffering condition (Fig 3B), assuming SK-channel half-activation, *K*_s_ = 0.33*μ*M [40]. Since EPSPs are less effective at activating VGCCs than NMDARs, the buffer-insensitive coupling distance for SK-channels and VGCCs was smaller (∼70nm) (Fig 3B). The reverse was true when a single bAP was considered (S4 Fig). These data show that, when SK-channels are tightly coupled to a Ca^2+^ source, Ca^2+^ buffering has limited effect on their activation.

**Fig 4:**
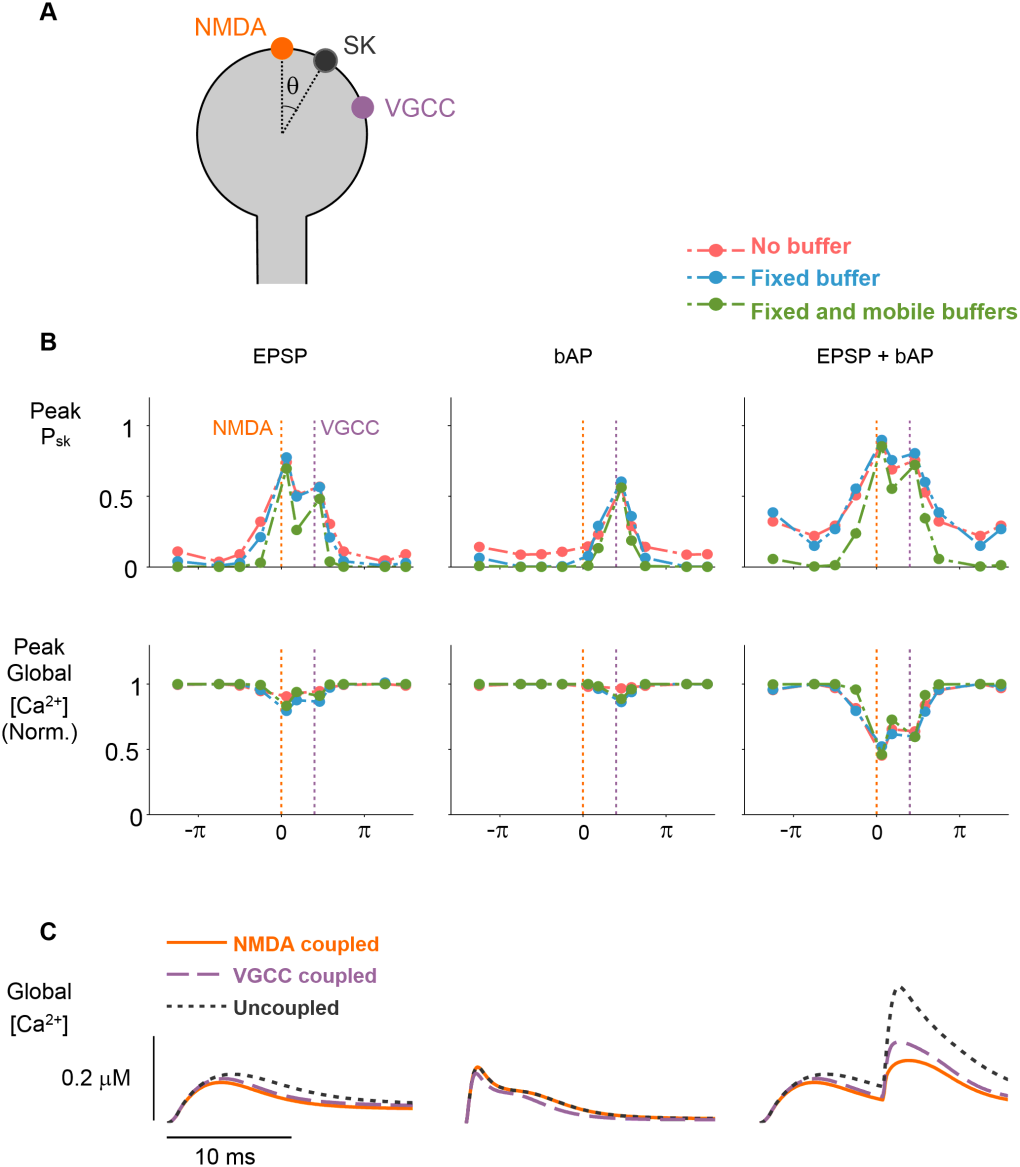
[Ca^2+^] transients elicited by compound stimuli, but not unitary stimuli, are sensitive to SK-channel activation. *(A)* Schematic showing ion-channel locations during SK-position sweep simulations. NMDAR and VGCC Ca^2+^ sources were fixed. SK-channel cluster position (defined by angular coordinate, *θ*) was varied. *(B)* Peak SK-activation, P_sk_, is sensitive to SK-channel location across all three stimulation patterns. For short SK-channel/Ca^2+^ -source coupling-distances peak SK-activation is high, whereas larger coupling distances result in low SK-activation (top row). Peak global [Ca^2+^] is sensitive to SK-channel location during application of compound stimuli but not unitary stimuli (bottom row). *π* radians is equivalent to ≈1.6 *μ*m along the membrane. *(C)* Global [Ca^2+^] -transients elicited by three different stimuli across three conditions for SK-channel coupling. SK-channel to Ca^2+^ source distance is 50nm for coupled conditions. For uncoupled condition, SK-channel is located on opposite side of spine head. Note that the enhancement of the global [Ca^2+^] transient is only seen during the compound stimulus in the uncoupled SK-channel condition (gray trace).

Next, we varied the position of the SK-channel cluster, defining its angular coordinate, *θ*, as a free parameter (Fig 4A). For all stimulation protocols, peak SK-channel activation was greatest when the SK-channel cluster was located near to an active Ca^2+^ source, and substantially reduced at increased coupling distances and Ca^2+^ buffering capacities (Fig 4B). During EPSP or bAP stimuli, peak global [Ca^2+^] was only weakly dependent on SK-channel to Ca^2+^ source coupling distance (Fig 4B). However, we observed a large inhibition of [Ca^2+^] transients evoked by the combined EPSP-bAP stimulation even though peak SK-activation was similar to that of single EPSP or bAP stimuli. This apparently contradictory result could be resolved by closer inspection of the global [Ca^2+^] transient. This showed that the effect of SK-channel activation was largely restricted to the [Ca^2+^] response to the bAP occurring after the EPSP (Fig 4C). This effect, and the weak influence of SK-channel activation on single EPSP or bAP stimulus, can be explained by the speed of SK-channel activation (*τ*_s_ ∼ 6ms) [40], which is slow relative to NMDAR and VGCC activation during unitary stimuli (Fig 3A). During the compound EPSP-bAP protocol, the initial priming stimulus, the EPSP, activates SK-channels that are spatially coupled to either Ca^2+^ source. The SK-channel activation time constant is too slow to influence the initial EPSP-elicited Ca^2+^ current, but for stimuli that occur shortly after the priming EPSP—in this case a bAP with 10ms delay—SK-activation has a significant influence over [Ca^2+^] levels. Furthermore, this priming effect on SK-channels was largely insensitive to the Ca^2+^ buffering capacity (Fig 4B).

These results demonstrate that SK-channel activation has a powerful influence over [Ca^2+^] transients elicited by combinations of stimuli, but not for [Ca^2+^] transients elicited by unitary stimuli. These results also suggest that SK-channels only influence global [Ca^2+^] when they are situated within ∼200nm of a Ca^2+^ source (Fig 4B), therefore, for SK-channels to have any function in Ca^2+^ -dependent synaptic-plasticity, they must be organized such that they are in close proximity to a Ca^2+^ source.

### Calmodulin is a sophisticated [Ca^2+^] -signal detector

CaM activation by spine Ca^2+^ signals is required for the expression of LTP. CaM is fully activated when its four Ca^2+^ binding sites are occupied. Two of these sites are found on its N-lobe and two on its C-lobe, each lobe demonstrating different affinities and kinetics [14, 25]. This leads to a highly non-linear relationship between [Ca^2+^] and activated CaM that potentially explains discrepancies between the calcium hypothesis for the induction of synaptic plasticity and experimental data [41]. To test this possibility, we next asked the following two questions: Do plasticity-inducing firing patterns (such as the canonical LTP-inducing EPSP-bAP) result in higher global concentrations of activated CaM in the spine? Do plasticity-inducing protocols produce CaM signals that exhibit particular spatial or time-dependent signatures which more benign firing patterns (such as unitary stimuli) do not?

In order to address these questions we investigated the spatiotemporal patterns of Ca^2+^ -bound CaM concentration ([CaCaM]) generated in the spine in response to both unitary and compound stimuli. In particular, we focused on a STDP induction protocol—a single EPSP followed by two bAPs at 10ms intervals (EPSP-2bAPs)—which is associated with robust LTP at the Schaffer collateral synapse in CA1 of the hippocampus [42, 43]. We were also interested to see how the CaM N- and C- lobes, with their distinct binding properties, responded to the different stimuli. To achieve this, we analyzed the responses of three different types of CaCaM species in the spine: total fully-activated CaCaM with all four Ca^2+^ -binding sites occupied; total CaCaM with fully-occupied N-lobe; and total CaCaM with fully-occupied C-lobe. In these simulations, fixed and mobile buffers were included and SK-channels were spatially-coupled to the NMDAR cluster (coupling distance of 50nm).

**Fig 5:**
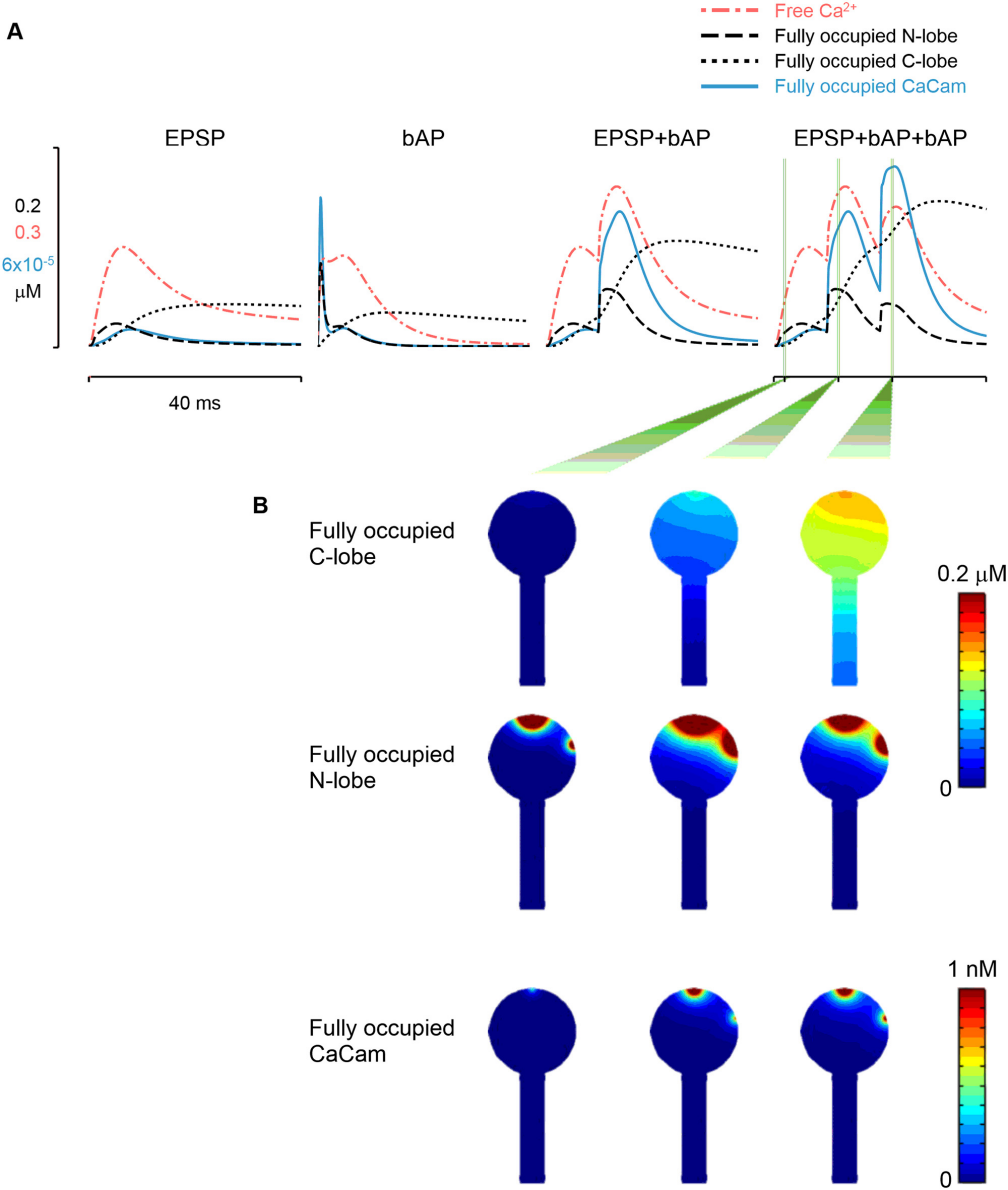
The combined responses of CaM N- and C- lobes to [Ca^2+^] result in a sophisticated Ca^2+^ sensor. *(A)* Globally-averaged time-courses of [Ca^2+^] and three CaCaM-species concentrations during four stimulation patterns. The high-affinity C-lobe integrates [Ca^2+^] signals over multiple stimuli (black dotted traces). The faster but low-affinity N-lobe demonstrates full-occupancy (black dashed traces) only during high [Ca^2+^] (red traces). The combined effect produces a peak amplitude CaCaM signal (blue traces) that increases with bAP number following an EPSP, whilst the timing of the peaks preserves time-delayed temporal information from the original [Ca^2+^] signal. *(B)* 2-dimensional cut-slice representation of three CaCaM-species concentrations. Fully-occupied C-lobe concentration equilibrates across spine due to high affinity for Ca^2+^ (top row). High levels of fully-occupied N-lobe are restricted to the immediate vicinity of Ca^2+^ sources due to the N-lobe’s fast binding kinetics and low affinity for Ca^2+^ (middle row). As a consequence, fully-activated CaCaM is also restricted to channel nanodomains (bottom row).

[CaCaM] and levels of fully-occupied CaM N-lobe demonstrated a strong spatial dependence with highest levels found in the immediate vicinity of the Ca^2+^ channel clusters (Figs 5, S5 and S6). This was due to the low affinity of the CaM N-lobe for Ca^2+^. The spatial dependence of the fully-occupied, high-affinity C-lobe was much weaker than that shown by the N-lobe. The C-lobe’s high affinity for Ca^2+^ resulted in a bound-state time-period sufficient for diffusion to equilibrate fully-occupied C-lobe levels across the spine (Figs 5 and S5) [25]. Thus, the diffusion of partially activated CaCaM (i.e., C-lobe occupied CaM) provides a means of communication between spine Ca^2+^ signaling nanodomains.

Globally, [Ca^2+^] signals were effectively integrated by the level of Ca^2+^ -occupied CaM C-lobe, whereas the level of Ca^2+^ -occupied CaM N-lobe—which has a lower affinity but much faster forward-binding rate than the CaM C-lobe—tracked the [Ca^2+^] signal in time (Figs 5 and S5). Fully-activated [CaCaM] amplitude and time integral were largest for the EPSP-2bAPs induction protocol (Figs 5 and S5). Furthermore, following the initial EPSP, subsequent [CaCaM] peaks increased in amplitude with the number of postsynaptic stimuli. From this we conclude that the distinct Ca^2+^-binding properties of the two CaM lobes give the CaM molecule the properties of a sophisticated Ca^2+^-sensor, suited to detecting the precise timing and number of post-synaptic spikes following an EPSP, as well as providing a communication channel between Ca^2+^ signalling nanodomains.

### SK-channels are powerful modulators of CaM activation in dendritic spines

**Fig 6:**
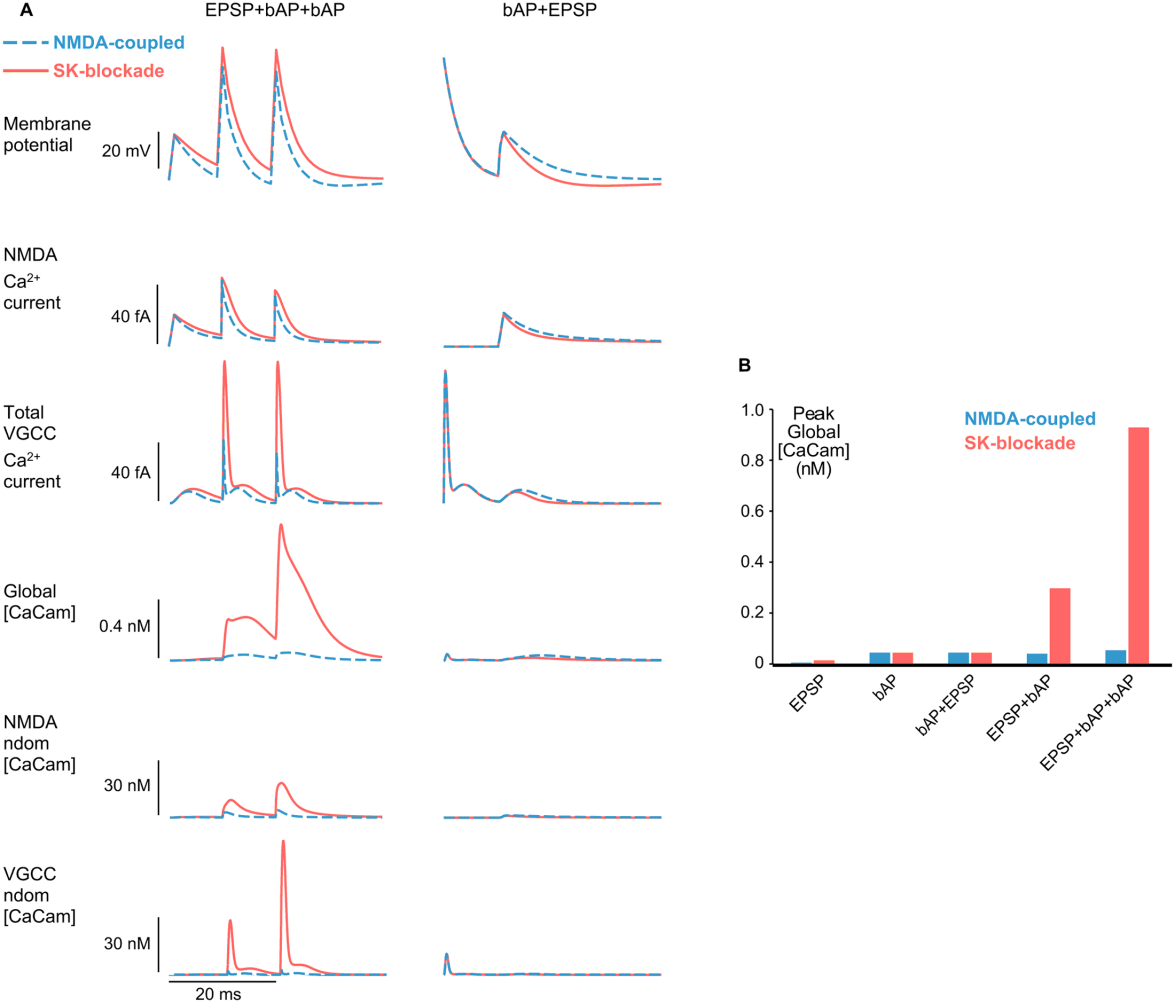
SK-channel blockade enhances CaM activation during LTP-inducing stimuli. *(A)* SK-channel blockade enhances spine membrane depolarization, in particular during bAPs, which substantially increases activation of HVA L-type VGCCs (EPSP-2bAPs, third panel down). The additional Ca^2+^ -influx results in an order of magnitude increase in fully-activated CaCaM both globally and in channel nanodomains. The effect is not observed in the bAP-EPSP protocol since there is no SK-priming presynaptic stimulus before the postsynaptic spike. *(B)* Bar chart summarizing SK-blockade effect on peak global [CaCaM] levels across different stimuli. [CaCaM] enhancement by SK-blockade is greatest for compound stimuli with the form of STDP protocols which classically induce LTP.

SK-channel inhibition facilitates learning and spatial memory formation [44], and modulates the induction of synaptic plasticity [19]. Since CaM activation is the first step in the signaling-cascades that lead to LTP, we investigated the effect of blocking SK-channels on [CaCaM] in dendritic spines during different stimuli. We examined the effect across multiple induction protocols (S8 Fig) but in particular focused on two STDP induction protocols, EPSP-2bAPs and bAP-EPSP (Fig 6).

During the EPSP-2bAPs protocol, blockade of SK-channels increased membrane potential depolarization leading to larger NMDAR and VGCC Ca^2+^ currents (Fig 6A). In particular, during the two bAPs SK-channel blockade substantially increased the peak VGCC Ca^2+^ -current by specifically boosting HVA or L-type VGCC activation. (Fig 6A; HVA VGCC current increased by 222% following SK blockade). Most dramatically, SK-channel blockade resulted in a 17-fold increase in the peak global [CaCaM] signal. [CaCaM] signal amplitudes in the NMDAR and VGCC nanodomains were 5- and 24-fold higher respectively during SK blockade. [CaCaM] sensitivity to SK blockade was most pronounced in the VGCC nanodomain since SK-channel blockade enhanced bAP amplitudes over the threshold for HVA L-type VGCC activation; an effective switch causing a brief but large influx of Ca^2+^ into the VGCC nanodomain (Fig 6A). The increase in [CaCaM] with SK-channel blockade was a consistent observation across a range of HVA VGCC conductances (S9 Fig) indicating the qualitative effects of SK-channel blockade did not depend on the degree of VGCC clustering.

We also investigated the sensitivity of [CaCaM] to SK-channel blockade during other compound stimuli including the canonical LTP and LTD STDP protocols (EPSP-bAP and bAP-EPSP respectively) as well as two EPSPs or two bAPs separated by 10ms (2EPSPs and 2bAPs respectively) (S8 Fig). For stimulation protocols that started with a bAP (2bAPs or bAP-EPSP), [CaCaM] response was very small and largely insensitive to SK-channel blockade. [CaCaM] during the 2EPSPs protocol was sensitive to SK-channel blockade but [CaCaM] was still low in comparison to the LTP inducing EPSP-2bAPs protocol. Interestingly, although SK-channel blockade enhanced [CaCaM] in response to EPSP-bAP stimulation (peak global [CaCaM] increased by 7-fold) the enhancement was much greater for EPSP-2bAPs stimulation (global peak [CaCaM] increased 17-fold) (Figs 6B and S8) thereby providing a mechanism for the requirement of bursts of bAPs at Schaffer collateral synapses in the hippocampus during STDP. Our results strongly support the conclusion that SK-channel inhibition has a powerful modulatory effect on [CaCaM] during neural firing patterns that are associated with LTP.

## Discussion

Our analyses show that SK-channels may play a central regulatory role in the induction of Hebbian synaptic-plasticity. 3-dimensional modeling of spine Ca^2+^ and CaM dynamics revealed that NMDAR-coupled SK-channels potentially modulate activated [CaCaM] up to 17-fold during STDP induction protocols associated with LTP, indicating that SK-channels effectively ‘gate’ the induction of STDP. Our results suggest that the mechanism behind this is ‘priming’ of SK-channels during EPSPs, by NMDAR activation, which effectively silence L-type VGCCs during subsequent bAPs. L-type VGCCs then provide a crucial Ca^2+^ signal for CaM (and therefore CaMKII) activation when SK-channels are inhibited. We have also shown that, in tandem, the distinctive Ca^2+^-binding properties of the CaM N- and C-lobes give the CaM molecule some sophisticated Ca^2+^ sensing properties.

3-dimensional modelling improves the biological plausibility and validity of our analyses. There is strong evidence to suggest that Ca^2+^ signal location in spines is an important determinant for synaptic plasticity, or—at the very least—that the amplitude and temporal characteristics of the spine Ca^2+^ signal is not sufficient to explain all plasticity observations. Many studies of spine Ca^2+^ signalling have concluded that the spatial localisation of Ca^2+^ sources is important for the ultimate biological role of the Ca^2+^ signal (see [45] for a review). It is essential to use a 3-dimensional model in order to allow for resolving spatially the Ca^2+^ sources and thus investigating this possibility. We also wished to investigate the effect of SK-channel location relative to NMDAR and VGCC Ca^2+^ sources, which again entails using a 3-dimensional spatial model. 3-dimensional modelling has been used successfully before to investigate the Ca^2+^/CaM interaction in the spine [33, 46]. Several of our findings depend on the spatial restriction of Ca^2+^/CaCaM signals (for example the difference in CaCaM activation in response to the relative timing of pre- and post-synaptic activation) showing that 3-dimensional modelling is capable of revealing roles of Ca^2+^ signalling that would be missed using a non-spatial model.

Endogenous fixed and mobile Ca^2+^ buffers reduce the amplitude of global [Ca^2+^] signals whereas nanodomain [Ca^2+^] signals are less affected. In particular, during bAPs, fast [Ca^2+^] spikes are absent from the global signal due to Ca^2+^ buffering. Thus, endogenous Ca^2+^ buffers act much like a low-pass filter for global [Ca^2+^] signals [33]. This filtering becomes less pronounced as the [Ca^2+^] signal readout location approaches a Ca^2+^ source meaning that the endogenous Ca^2+^ buffers will have a limited effect on CaM activation when CaM is located within channel nanodomains. Previous theoretical work using 3-dimensional simulation of Ca^2+^ signalling has demonstrated that CaM activation at nanodomain is affected by its diffusion [46]. We have tested this in our model by varying the diffusivity of CaM. CaM activation in the nanodomain varies by degree with CaM diffusivity as predicted by Naoki et al. [46] (see S7A Fig). However, these simulations also reveal that the main conclusion of the paper, i.e. the modulation of CaM activation by SK channels, is still applicable across the range of diffusivities tested (see S7B & S7C Fig). Endogenous Ca^2+^ buffering prevents the crosstalk of [Ca^2+^] signals between NMDA and VGCC nanodomains, however, communication between nanodomains could be enabled by C-lobe occupied CaM which disperses almost uniformly throughout the spine head [25]. Because of the dependence of CaM activation on nanodomain Ca^2+^ signalling, spine morphology is not predicted to have a major impact on CaM activation although this remains to be tested.

In the classic Hebbian stimulation for LTP induction—an EPSP followed by a bAP—EPSP-elicited Ca^2+^ influx will create a pool of C-lobe occupied CaM that diffuses into VGCC nanodomains ready for the N-lobe to be populated by subsequent high influx of Ca^2+^ through HVA L-type VGCCs. Interestingly, the reverse sequence of stimuli is not so effective at generating fully-activated [CaCaM] because bAPs preferentially generate N-lobe occupied CaM which unbinds Ca^2+^ rapidly and therefore is not capable of integrating over compound stimuli [34, 47]. The importance of L-type VGCCs for CaM activation is supported by the observation that CaMKII activation is abolished during blockade of L-type VGCCs [48].

Given the highly effective endogenous buffering of Ca^2+^, if SK-channels are to influence spine membrane potential and Ca^2+^ influx, then they must be spatially coupled to a Ca^2+^ source (Fig 4A). SK-channels have been reported to be co-located with both VGCCs and NMDARs in dendritic spines [17, 29, 39]. Our data suggest SK-channels are equally activated during EPSPs when coupled to NMDARs or LVA VGCCs since the EPSP depolarization is sufficient to activate T-type VGCCs. Hence, SK-channels coupled to LVA VGCCs will be ‘primed’ by EPSPs in much the same way as those coupled to NMDARs. An important property of SK-channels is their relatively slow activation (*τ*∼ 6ms) [40] which leads to the different [Ca^2+^] responses to unitary and compound stimuli. SK-channel activation following a single bAP is too slow for the inhibitory current to influence the fast-activating HVA L-type VGCC Ca^2+^ current. However, if a bAP occurs shortly after an initial ‘priming’ stimulus, then the SK-current effectively silences the L-type Ca^2+^ channels by repolarizing the membrane potential below the threshold voltage for L-type VGCC activation. Interestingly, fully-activated [CaCaM] signals were insensitive to SK-channel blockade for induction protocols not associated with plasticity (S8 Fig). On the other hand, for protocols involving an EPSP followed by 1 or more bAPs (the form of the classic STDP LTP-inducing protocols), SK-channel blockade enhanced fully-activated [CaCaM] by an order of magnitude. This suggests a potential gating mechanism for the induction of STDP, which could be potentially tested using a FRET based CAMKII sensor [48].

Strong clustering of VGCCs will increase the local influx of Ca^2+^ thereby saturating endogenous buffers, which will have an effect on other Ca^2+^ interactions. Strong vs. weak clustering of channels could be viewed as effectively altering the VGCC ‘cluster’ conductance. We probe the effect of varying VGCC cluster conductance on the activation of CaCaM (see S9A Fig) and find that across the conductance range tested, dramatic changes in [CaCaM] activation are still seen with SK-blockade (see S9B Fig). This is due to the steep sigmoidal dependence of L-type VGCC activation on membrane voltage, which is independent of VGCC conductance. We have also simulated the case where the VGCC conductance is uniform over the spine surface. The effect of changing the VGCC distribution from clustered to uniform is to reduce the global levels of [CaCaM] in both SK-channel conditions although there is still substantial [CaCaM] when VGCCs are distributed uniformly (S10 Fig). As expected, in the VGCC nanodomain (which is defined in both conditions as 20nm radial distance from the VGCC Ca^2+^ source in the cluster condition), the [CaCaM] signal is greatly reduced by changing the spatial distribution of the VGCC conductance. In a sense, when the VGCC current density is uniform over the entire surface of the spine, there is no VGCC nanodomain. In the case of uniform distribution of VGCCs the main conclusion of the paper, that SK-channels are powerful modulators of CaM activation still holds.

SK-channels are negatively regulated by neuromodulators such as acetylcholine and noradrenaline [18, 49, 50] and by metabotropic glutamate receptors [9]. Furthermore, LTP is facilitated by activating M1 muscarinic or metabotropic receptors respectively, which both inhibit SK-channels leading to enhanced NMDAR activation during STDP induction [9, 18]. Our results propose an additional mechanism by which neuromodulators that inhibit SK-channels could gate LTP by unsilencing the L-type VGCC [Ca^2+^] signal during STDP LTP induction protocols leading to greatly enhanced CaM (and therefore CaMKII) activation. Thus, SK-channels may provide a common mechanism by which multiple neuromodulators can gate Hebbian plasticity enabling the switching of neuronal networks, such as the hippocampus, between LTP-competent and LTP-incompetent configurations.

## Supporting Information

S1 Table Computational model details and parameter values. (PDF)

S1 Fig Reaction network of Ca^2+^ /calmodulin co-operative binding. N_*x*_ and C_*x*_, represent calmodulin N- and C-lobe Ca^2+^ binding-sites. Binding sites are initially in state T, with associated binding rates *k*_on(T)*x*_ and *k*_off(T)*x*_. Upon binding Ca^2+^, the lobe’s remaining Ca^2+^ binding site instantaneously switches to R state, with associated binding rates *k*_on(R)*x*_ and *k*_off(R)*x*_.
(TIF)

S2 Fig Low-pass filtering of global [Ca^2+^] signals by endogenous Ca^2+^ buffers. Additional stimuli for Fig 2. Nanodomain [Ca^2+^] is defined as Ca^2+^ concentration at 50nm radial distance from channel cluster.
(TIF)

S3 Fig Ca^2+^ buffer parameter sweeps. Comparison of global and nanodomain [Ca^2+^] transients for EPSP+bAP stimulation protocol. Nanodomain [Ca^2+^] is defined as [Ca^2+^] at 20nm radial distance from channel cluster. Simulations use the simple binding scheme for Ca^2+^ to mobile Ca^2+^ buffer described in equation system 17. The first two columns show transients for simulations where EFB parameters were fixed and the concentration of the mobile buffer was varied. Mobile Ca^2+^ buffer parameter values were selected to represent a typical ‘fast’ mobile buffer and a ‘slow’ mobile buffer. Slow mobile buffer binding parameters were set to match estimates for calbindin [25] and fast mobile buffer was set to CaM N-lobe binding rates [51]. The end two columns show transients for simulations where the mobile Ca^2+^ buffer was set to 100*μ*M calbindin and either the EFB binding forward rate or EFB affinity was fixed while the other varied.
(TIF)

S4 Fig *(A)* Peak [Ca^2+^] gradients in Ca^2+^ channel nanodomains across buffer condition and spine stimuli. *(B)* Peak [Ca^2+^] gradients in Ca^2+^ channel nanodomains in the presence of EFB and 100*μ*M calbindin. Each curve represents a different stimulation pattern. Dashed horizontal lines in all plots indicate SK half-activation parameter, *K*_s_ = 0.33*μ*M.
(TIF)

S5 Fig Global and nanodomain (20nm from channel cluster) time courses of [Ca^2+^] and CaCaM species concentrations across different spine stimuli. SK-channels were active and spatially coupled (50nm) to NMDAR cluster.
(TIF)

S6 Fig Peak CaCaM concentration profiles in channel nanodomains across different stimuli. SK-channels were active and spatially coupled (50nm) to NMDAR cluster.
(TIF)

S7 Fig *(A)* Concentration gradients of peak activated [CaCaM] in NMDA and VGCC nanodomains during EPSP-bAP induction protocols. Each curve represents different simulated values for CaM diffusivity. *(B)* Activated [CaCaM] is increased by SK-blockade across CaM diffusivities. Very immobile CaM results in heightened effect of SK-blockade on activated [CaCaM] (see also C). *(C)* Ratio of peak [CaCaM] for SK-channel conditions. Subscript 0 indicates that SK-channels were blocked and subscript 1 indicates SK-channels were active.
(TIF)

S8 Fig [CaCaM] transients elicited by classical LTP-inducing STDP protocols are sensitive to SK-blockade whereas other compound and unitary stimuli are not. EPSPs were required for SK-channel activation since SK-channel cluster was spatially coupled to NMDAR cluster (50nm) for these simulations. Bar charts in end column summarize global and nanodomain peak [CaCaM].
(TIF)

S9 Fig *(A)* Concentration gradients of peak activated [CaCaM] in NMDA and VGCC nanodomains during EPSP-bAP induction protocols. Each curve represents different simulated values for L-type channel cluster conductance. *(B)* Activated [CaCaM] is increased by SK-blockade across a range of L-type conductances although differences are enhanced as L-type conductance is increased (see also C). *(C)* Ratio of peak [CaCaM] for SK-channel conditions blocked or coupled to NMDARs. Subscript 0 indicates that SK-channels were blocked and subscript 1 indicates SK-channels were active.
(TIF)

S10 Fig The effect of SK-blockade on [CaCaM] transients elicited by EPSP-bAP protocol for two VGCC spatial arrangements. Clustered VGCC condition refers to the VGCC arrangement described in the main text and illustrated in Fig 1. Uniform VGCC condition refers to the case where the total VGCC conductance is spread uniformly across the entire surface of the spine. VGCC nanodomain, for both spatial arrangements of VGCCs, is defined as a point 20nm radial distance from the VGCC Ca^2+^—source in the clustered case.
(TIF)
