# Supplementary material for: Control of Ca2+ Influx and Calmodulin Activation by SK-Channels in Dendritic Spines
Source: PLoS Comput Biol. 2016 May 27;12(5):e1004949. doi: 10.1371/journal.pcbi.1004949 (PMC4883788; doi:10.1371/journal.pcbi.1004949)
Supplement: S1 Table — (PDF) [file pcbi.1004949.s001.pdf]

Supporting Information: Control of Ca^{2+} influx and calmodulin activation by SK-channels in dendritic spines

Thom Griffith [1,2], Krasimira Tsaneva-Atanasova [3,4,*,‡], Jack R. Mellor [5,*,‡]

1 Department of Engineering Mathematics, University of Bristol, Bristol, BS8 1UB, UK
2 Bristol Centre for Complexity Sciences, University of Bristol, Bristol, BS2 8BB, UK
3 Department of Mathematics, College of Engineering, Mathematics and Physical Sciences, University of Exeter, Exeter, EX4 4QF, UK
4 EPSRC Centre for Predictive Modelling in Healthcare, University of Exeter, Exeter, EX4 4QJ, UK
5 Centre for Synaptic Plasticity, School of Physiology, Pharmacology and Neuroscience, University of Bristol, Bristol, BS8 1TD, UK

‡The senior authors also contributed equally to this work
*** Jack.Mellor@Bristol.ac.uk or K.Tsaneva-Atanasova@exeter.ac.uk**

Model equations

In our model, NMDAR- and VGCC-dependent spine-calcium influx varies in response to pre- and postsynaptic spiking activity, the spike-timings of which act as the model input. Spine membrane potential, V_{m}, is the sum of the membrane depolarization due to synaptically-evoked currents, V_{i}, and back-propagating action potentials, V_{bAP}.

Back-propagating action potentials

Depolarization due to bAPs is modeled with a double exponential, as in [1, 2]

$$V_{\mathrm{bAP}}(t) = \sum_{\mathrm{post}} \left\{ V_{\max} \left[I_{\mathrm{b_f}} \exp\left(\frac{t_{\mathrm{post}} - t}{\tau_{\mathrm{b_f}}}\right) + I_{\mathrm{b_s}} \exp\left(\frac{t_{\mathrm{post}} - t}{\tau_{\mathrm{b_s}}}\right) \right] \right\}, \quad (1)$$

where $I_{\mathrm{b_s}}$ and $I_{\mathrm{b_f}}$ set the relative contributions of the fast and slow components and t_{post} is the array of all postsynaptic spike times. The total contribution to membrane depolarization is a summation over all t_{post}, so bAPs in close temporal proximity have an additive effect.

Ion currents

The model includes the following currents: I_{A} and I_{N} mediated by AMPARs and NMDARs, I_{CaT} and I_{CaL} mediated by T-type and L-type VGCCs, a calcium-activated K^{+} current, I_{SK}, mediated by SK-channels, and a leak current, I_{L}. VGCC currents are small relative to the other currents, so we assume negligible VGCC contribution to spine depolarization. VGCCs therefore act simply as voltage-dependent Ca^{2+} sources. Using Hodgkin-Huxley formalism, the spine membrane potential due to ion channel currents, V_{i}, evolves according to

$$C_{\mathrm{m}}\frac{dV_{\mathrm{i}}}{dt} = -I_{\mathrm{A}}(V_{\mathrm{m}}) - I_{\mathrm{N}}(V_{\mathrm{m}}) - I_{\mathrm{SK}}(V_{\mathrm{m}}, c) - I_{\mathrm{L}}(V_{\mathrm{m}}), \tag{2}$$

where C_{m} is the membrane capacitance and c is [Ca^{2+}] local to the SK-channel. The AMPAR, NMDAR and leak ion currents are modeled as

$$I_{\mathrm{A}}(V_{\mathrm{m}}) = -g_{\mathrm{A}} r_{\mathrm{A}}(t)\,(V_{\mathrm{m}} - E_{\mathrm{A}}) \tag{3}$$
$$I_{\mathrm{N}}(V_{\mathrm{m}}) = -g_{\mathrm{N}} r_{\mathrm{N}}(t) B_{\mathrm{N}}(V_{\mathrm{m}})\,(V_{\mathrm{m}} - E_{\mathrm{N}}) \tag{4}$$
$$I_{\mathrm{L}}(V_{\mathrm{m}}) = g_{\mathrm{L}}\,(V_{\mathrm{m}} - E_{\mathrm{L}})\,, \tag{5}$$

where r_A and r_N are the fraction of glutamate-bound receptors in the relevant synaptically-activated ion-channel cluster (calculated as in [3]), and $B_N(V_{\mathrm{m}})$ is a term representing NMDAR voltage-dependent Mg^{2+} unblock (fit from [4]), given as

$$B_{\mathrm{N}}(V_{\mathrm{m}}) = \left[1 + \frac{[\mathrm{Mg}^{2+}]}{3.57}\exp\left(\frac{-V_{\mathrm{m}}}{16.13}\right)\right]^{-1}. \tag{6}$$

The calcium-activated SK-current is given by,

$$I_{\mathrm{SK}}(V_{\mathrm{m}}, c) = g_{\mathrm{SK}} s\,(V_{\mathrm{m}} - E_{\mathrm{SK}}) \tag{7}$$
$$\frac{ds}{dt} = \frac{s_{\infty}(c) - s}{\tau_{\mathrm{s}}} \tag{8}$$
$$s_{\infty}(c) = \frac{c^n}{c^n + K_{\mathrm{s}}^n}, \tag{9}$$

where c is the local Ca^{2+} concentration at the SK-channel, τ_{s} is the SK-activation time constant, K_m is the half-activation parameter and n is the Hill coefficient [5].

Ca^{2+} fluxes

Ca^{2+} fluxes are calculated from the Ca^{2+} component of the NMDAR current and the VGCC currents. The Ca^{2+} component of the NMDAR current is given by

$$I_{\mathrm{Ca_N}}(V_{\mathrm{m}}) = (P_{\mathrm{f}}/3)\,g_{\mathrm{N}} r_{\mathrm{N}}(t) B_{\mathrm{N}}(V_{\mathrm{m}})(V_{\mathrm{m}} - E_{\mathrm{Ca}}), \tag{10}$$

where P_{f} is the fractional contribution of Ca^{2+} to the NMDAR current at -60mV, and E_{Ca} is the Ca^{2+} reversal potential. The additional correction factor of 1/3 is applied to P_{f}, due to the difference in reversal potentials for the NMDAR current Ca^{2+} component and total NMDAR current.

VGCC Ca^{2+} currents are modeled as in [6]

$$I_{\mathrm{Ca_T}}(V_{\mathrm{m}}) = g_{\mathrm{Ca_T}} m^2_{\mathrm{Ca_T}}(V_{\mathrm{m}}) h_{\mathrm{Ca_T}}(V_{\mathrm{m}})\,(V_{\mathrm{m}} - E_{\mathrm{Ca}}) \tag{11}$$
$$I_{\mathrm{Ca_L}}(V_{\mathrm{m}}) = g_{\mathrm{Ca_L}} m^2_{\mathrm{Ca_L}}(V_{\mathrm{m}}) h_{\mathrm{Ca_L}}(V_{\mathrm{m}})\,(V_{\mathrm{m}} - E_{\mathrm{Ca}})\,, \tag{12}$$

where the activation and inactivation gating variables take the form

$$\frac{d\sigma}{dt} = \frac{\sigma_{\infty}(V_{\mathrm{m}}) - \sigma}{\tau_{\sigma}}, \tag{13}$$

where $\sigma \in \{m_{\mathrm{Ca_T}}, h_{\mathrm{Ca_T}}, m_{\mathrm{Ca_H}}, h_{\mathrm{Ca_h}}\}$. The steady-state functions, σ_{∞}, are given by

$$\sigma = \frac{1}{1 + \exp\left[-\left(V_{\mathrm{m}} - V_{\sigma}\right)/k_{\sigma}\right]}. \tag{14}$$

All Ca^{2+} currents were converted to Ca^{2+} fluxes

$$J_{\upsilon} = \frac{I_{\upsilon}}{2F\Gamma_{\upsilon}}, \tag{15}$$

where $\upsilon \in \{\mathrm{N}, \mathrm{A}, \mathrm{Ca_T}, \mathrm{Ca_L}\}$, F is Faraday's constant, and Γ_{υ} is the surface-area on the spine boundary representing the channel cluster.

Ca^{2+} extrusion

The various Ca^{2+} extrusion mechanisms in the spine were modeled as a single linear term in Ca^{2+} concentration, c. The extrusion rate, γ, is constant and was estimated using Equation 4 in [7], and Ca^{2+} imaging data from [8] and [9].

Ca^{2+} buffering

For the simplest case (those simulations not involving the complex Ca^{2+}/calmodulin model), the binding of Ca^{2+} to buffer species, B, was modeled simply as

$$\mathrm{Ca}^{2+} + \mathrm{B} \underset{k^-}{\overset{k^+}{\rightleftharpoons}} \mathrm{CaB}, \tag{16}$$

with apparent forward and backward binding rates, k^+ and k^-.

Inclusion of the Ca^{2+} -extrusion term and assuming an unchanged D_{B} on binding of the buffer to the much smaller Ca^{2+} ion and uniform initial buffer concentration leads to the following reaction-diffusion system

$$\begin{aligned}
\frac{\partial c}{\partial t} &= D_{\mathrm{Ca}}\nabla^2 c - k_{\mathrm{b}}^{+} cb + k_{\mathrm{b}}^{-}(b_{\mathrm{tot}} - b) - k_{\mathrm{u}}^{+} cu + k_{\mathrm{u}}^{-}(u_{\mathrm{tot}} - u) - \gamma c \\
\frac{\partial b}{\partial t} &= D_{\mathrm{B}}\nabla^2 b - k_{\mathrm{b}}^{+} cb + k_{\mathrm{b}}^{-}(b_{\mathrm{tot}} - b) \\
\frac{\partial u}{\partial t} &= -k_{\mathrm{u}}^{+} cu + k_{\mathrm{u}}^{-}(u_{\mathrm{tot}} - u),
\end{aligned} \tag{17}$$

where c, b and u are the Ca^{2+}, mobile buffer, and EFB concentrations respectively.

EFB concentrations were calculated using the following relationship, which is valid when the resting Ca^{2+} concentration is taken as zero, as described in [10]

$$u_{\mathrm{tot}} = \kappa_{\mathrm{u}} K_{\mathrm{u}}, \tag{18}$$

where κ_{u} is the EFB binding ratio and K_{u} is the EFB affinity for Ca^{2+}.

Cooperative binding of Ca^{2+} to calmodulin

We used a detailed model of cooperative binding of Ca^{2+} to calmodulin [11]. The reaction network as described in [11] is shown in Supplementary Figure 1.
Ca^{2+}/calmodulin binding-scheme reaction-rates are the same as in [11].

Supplementary Table 1. Parameter values of the model (unless otherwise stated)

Notation	Parameter	Value	Source
$[Mg^{2+}]$	Extracellular Mg^{2+} concentration	1 mM	-
	Spine head diameter	0.52 μm	[12]
	Spine neck length	0.67 μm	[12]
	Spine neck diameter	0.15 μm	[12]
	Resting membrane potential	-65 mV	-
c_{m}	Specific membrane capacitance	0.009 pF μm^2	[13]
$\bar{g}_L$	Specific leak conductance	57.5 pS μm^{-2}	[14] **
$V_{\max}$	bAP maximum depolarization	67 mV	[15]
$I_{\mathrm{b_f}}$	Contribution of fast bAP component	0.99	[2]
$I_{\mathrm{b_s}}$	Contribution of slow bAP component	0.01	[2]
$\tau_{\mathrm{b_f}}$	Fast bAP time constant	3 ms	[1]
$\tau_{\mathrm{b_s}}$	Slow bAP time constant	25 ms	[1]
	AMPA glutamate forward binding rate	1100 s^{-1} mM^{-1}	[3]
	AMPA glutamate backward binding rate	190 s^{-1}	[3]
	NMDA glutamate forward binding rate	72 s^{-1} mM^{-1}	[3]
	NMDA glutamate backward binding rate	20 s^{-1}	[3]
$V_{\mathrm{m_{CaT}}}$	VGCC activation potential parameter	-54 mV	[6]
$k_{\mathrm{m_{CaT}}}$	VGCC activation parameter	5 mV	[6]
$\tau_{\mathrm{m_{CaT}}}$	VGCC activation time constant	2 ms	[6]
$V_{\mathrm{h_{CaT}}}$	VGCC inactivation potential parameter	-65 mV	[6]
$k_{\mathrm{h_{CaT}}}$	VGCC inactivation parameter	-8.5 mV	[6]
$\tau_{\mathrm{h_{CaT}}}$	VGCC inactivation time constant	32 ms	[6]
$V_{\mathrm{m_{CaL}}}$	VGCC activation potential parameter	-15 mV	[6]
$k_{\mathrm{m_{CaL}}}$	VGCC activation parameter	5 mV	[6]
$\tau_{\mathrm{m_{CaL}}}$	VGCC activation time constant	0.08 ms	[6]
$V_{\mathrm{h_{CaL}}}$	VGCC inactivation potential parameter	-60 mV	[6]
$k_{\mathrm{h_{CaL}}}$	VGCC inactivation parameter	-7 mV	[6]
$\tau_{\mathrm{h_{CaL}}}$	VGCC inactivation time constant	300 ms	[6]
g_{A}	AMPA conductance	60 pS	[16, 17]
g_{N}	NMDA conductance	160 pS	[16, 17]
g_{SK}	SK-channel conductance	25 pS	[18] **
$g_{\mathrm{Ca_T}}$	T-type VGCC conductance	0.23 pS	[19] **
$g_{\mathrm{Ca_L}}$	L-type VGCC conductance	0.9 pS	[19] **
E_{A}	AMPA reversal potential	0 mV	[3]
E_{N}	NMDA reversal potential	0 mV	[3]
E_{L}	Leak reversal potential	-65 mV	[3]
E_{Ca}	Calcium reversal potential	120 mV	[1]
E_{SK}	SK-channel reversal potential	-90 mV	-
τ_{s}	SK activation time constant	6.3 ms	[20]
K_{s}	SK gating half-activation parameter	0.33 μM	[20]
n	SK gating Hill coefficient	4	[20]
P_{f}	Frac. cont. of Ca^{2+} to I_{N} at -60mV	0.135	[21]
D_{Ca}	Diffusivity of Ca^{2+}	220 $\mu m^2 s^{-1}$	[22, 23]
D_{B}	Diffusivity of mobile Ca^{2+} buffer	20 $\mu m^2 s^{-1}$	[23]
γ	Ca^{2+} extrusion rate	5000 s^{-1}	[8, 9] **
κ_{u}	EFB binding ratio	20	[8]
u_{tot}	EFB concentration	2 mM	[8]
k_{u}^{+}	Physiological EFB forward binding rate	100 $\mu M^{-1} s^{-1}$	[24]
K_{u}	Physiological EFB dissociation constant	100 μM	[24]
b_{tot}	Mobile buffer concentration	100 μM	[11]

** Parameter was tuned to obtain best match to experimental data from the referenced source.

References

1. Shouval HZ, Bear MF, Cooper LN. A unified model of NMDA receptor-dependent bidirectional synaptic plasticity. Proceedings of the National Academy of Sciences of the United States of America. 2002;99(16):10831–6.

2. Rackham OJL, Tsaneva-Atanasova K, Ganesh A, Mellor JR. A Ca-Based Computational Model for NMDA Receptor-Dependent Synaptic Plasticity at Individual Post-Synaptic Spines in the Hippocampus. Frontiers in synaptic neuroscience. 2010;2:31.

3. Destexhe A, Mainen ZF, Sejnowski TJ, Torrey N, Road P, Jotia L, et al. Synthesis of models for excitable membranes, synaptic transmission and neuromodulation using a common kinetic formalism. Journal of computational neuroscience. 1994;1(3):195–230.

4. Jahr CE, Stevens CF. A Quantitative Description of NMDA Receptor-Channel Kinetic Behavior. The Journal of Neuroscience. 1990;10(6):1830–1837.

5. Chay TR, Keizer J. Theory of the effect of extracellular potassium on oscillations in the pancreatic beta-cell. Biophysical journal. 1985;48(5):815–27.

6. Nowacki J, Osinga HM, Brown JT, Randall AD, Tsaneva-Atanasova K. A unified model of CA1/3 pyramidal cells: an investigation into excitability. Progress in biophysics and molecular biology. 2011;105(1-2):34–48.

7. Helmchen F, Imoto K, Sakmann B. Ca2 + Buffering and Action Potential-Evoked Ca2 + Signaling in Dendrites of Pyramidal Neurons. Biophysical Journal. 1996;70:1069–1081.

8. Sabatini BL, Oertner TG, Svoboda K. The life cycle of Ca(2+) ions in dendritic spines. Neuron. 2002;33(3):439–52.

9. Cornelisse LN, van Elburg RaJ, Meredith RM, Yuste R, Mansvelder HD. High speed two-photon imaging of calcium dynamics in dendritic spines: consequences for spine calcium kinetics and buffer capacity. PloS one. 2007;2(10):e1073.

10. Neher E, Augustine GJ. Calcium gradients and buffers in bovine chromaffin cells. The Journal of physiology. 1992;450:273–301.

11. Faas GC, Raghavachari S, Lisman JE, Mody I. Calmodulin as a direct detector of Ca2+ signals. Nature neuroscience. 2011;14(3):301–4.

12. Tonnesen J, Katona G, Rózsa B, Nägerl UV. Spine neck plasticity regulates compartmentalization of synapses. Nature neuroscience. 2014;17(5):678–85.

13. Gentet LJ, Stuart GJ, Clements JD. Direct measurement of specific membrane capacitance in neurons. Biophysical journal. 2000;79(1):314–320.

14. Harnett MT, Makara JK, Spruston N, Kath WL, Magee JC. Synaptic amplification by dendritic spines enhances input cooperativity. Nature. 2012;491(7425):599–602.

15. Palmer LM, Stuart GJ. Membrane potential changes in dendritic spines during action potentials and synaptic input. The Journal of neuroscience : the official journal of the Society for Neuroscience. 2009;29(21):6897–903.

16. Racca C, Stephenson Fa, Streit P, Roberts JD, Somogyi P. NMDA receptor content of synapses in stratum radiatum of the hippocampal CA1 area. The Journal of neuroscience : the official journal of the Society for Neuroscience. 2000;20(7):2512–22.

17. Traynelis SF, Wollmuth LP, Mcbain CJ, Menniti FS, Vance KM, Ogden KK, et al. Glutamate Receptor Ion Channels : Structure , Regulation , and Function. Pharmacological Reviews. 2010;62(3):405–496.

18. Ngo-Anh TJ, Bloodgood BL, Lin M, Sabatini BL, Maylie J, Adelman JP. SK channels and NMDA receptors form a Ca2+-mediated feedback loop in dendritic spines. Nature neuroscience. 2005;8(5):642–9.

19. Bloodgood BL, Sabatini BL. Nonlinear regulation of unitary synaptic signals by CaV(2.3) voltage-sensitive calcium channels located in dendritic spines. Neuron. 2007;53(2):249–60.

20. Xia XM, Fakler B, Rivard A, Wayman G, Johnson-Pais T, Keen JE, et al. Mechanism of calcium gating in small-conductance calcium-activated potassium channels. Nature. 1998;395(6701):503–7.

21. Burnashev N, Zhou Z, Neher E, Sakmann B. Fractional calcium currents through recombinant GluR channels of the NMDA, AMPA and kainate receptor subtypes. The Journal of physiology. 1995;485 (Pt 2(1995):403–18.

22. Allbritton NL SL Tobias M. Range of messenger action of calcium ion and inositool 1,4,5-triphosphate. Science. 1992;258:1812–1812.

23. Naraghi M, Neher E. Linearized buffered Ca2+ diffusion in microdomains and its implications for calculation of [Ca2+] at the mouth of a calcium channel. The Journal of neuroscience : the official journal of the Society for Neuroscience. 1997;17(18):6961–73.

24. Nakamura Y, Harada H, Kamasawa N, Matsui K, Rothman JS, Shigemoto R, et al. Nanoscale Distribution of Presynaptic Ca2+ Channels and Its Impact on Vesicular Release during Development. Neuron. 2014;85(1):145–158.

25. Kubota Y, Putkey Ja, Waxham MN. Neurogranin Controls the Spatiotemporal Pattern of Postsynaptic Ca2+/CaM Signaling. Biophysical Journal. 2007;93(11):3848–3859.
